# Homoharringtonine exerts anti-silicosis effects by inhibiting the CCR1 and PI3K/AKT signaling pathways in lung fibroblasts

**DOI:** 10.7555/JBR.39.20250074

**Published:** 2025-05-21

**Authors:** Xinying Jia, Ziwei Li, Xiyue Hu, Ting Wang, Wenxiu Lian, Wenqing Sun, Yi Liu, Chunhui Ni

**Affiliations:** 1 Department of Occupational Medicine and Environmental Health, Key Laboratory of Modern Toxicology of Ministry of Education, School of Public Health, Nanjing Medical University, Nanjing, Jiangsu 211166, China; 2 Department of Public Health, Kangda College of Nanjing Medical University, Lianyungang, Jiangsu 222000, China; 3 Community Health Service Center of Anzhen Street, Xishan District, Wuxi, Jiangsu 214035, China; 4 Department of Pathology, Nanjing Drum Tower Hospital, the Affiliated Hospital of Nanjing University Medical School, Nanjing, Jiangsu 210000, China; 5 The Affiliated Wuxi Center for Disease Control and Prevention of Nanjing Medical University, Wuxi Center for Disease Control and Prevention, Wuxi Medical Center, Nanjing Medical University, Wuxi, Jiangsu 214400, China

**Keywords:** silicosis, homoharringtonine, lung fibroblast, PI3K/AKT/mTOR signaling pathway, CCR1

## Abstract

Silicosis is an occupational lung disease caused by prolonged exposure to silica dust in the workplace. It has a complex pathogenesis and currently lacks effective treatments. Homoharringtonine (HHT) is a natural compound approved for the treatment of acute myeloid leukemia, but its effects on silicosis remain unclear. In the present study, we constructed a mouse model of silica (SiO_2_)-induced pulmonary fibrosis and evaluated the preventive and therapeutic effects of HHT. The results showed that HHT significantly attenuated the progression of SiO_2_-induced pulmonary fibrosis in mice. We then used MRC-5, a human lung fibroblast cell line, to explore the mechanisms underlying HHT's inhibitory effects *in vitro* and found that HHT significantly inhibited the activation and migratory capacity of MRC-5 cells. Mechanistically, these effects were mediated by enhanced ubiquitination and degradation of the CCR1 protein. Furthermore, HHT exhibited favorable biocompatibility *in vivo*, and its preventive and therapeutic effects were validated in SiO_2_-treated mice. Collectively, the current study demonstrates that HHT shows significant potential as a therapeutic agent for silicosis by targeting CCR1 and the PI3K/AKT/mTOR signaling pathway, highlighting it as a promising candidate for clinical translation for silicosis treatment.

## Introduction

Silicosis is a major occupational interstitial lung disease caused by prolonged exposure to silica dust in the workplace, representing a significant global health issue^[1]^. Inhalation of silica (SiO_2_) damages the lung parenchyma, leading to inflammation, the formation of silicotic nodules, and progressive fibrosis^[2]^. Although patients may initially be asymptomatic, the disease gradually progresses to severe respiratory symptoms and chronic lung damage^[3]^. Silicosis remains an intractable public health problem in both developed and developing countries because of the lack of effective treatments, which underscores the urgent need for novel therapeutic strategies.

The pathogenesis of silicosis is complex and not yet fully understood, involving multiple cell types. Among these, lung fibroblasts are key effector cells in disease progression^[4]^. Upon activation by cytokines, fibroblasts differentiate into myofibroblasts, which secrete excessive extracellular matrix, ultimately causing pulmonary fibrosis^[5]^. Notably, pharmacological blockade of the CD44-RhoA-YAP signaling pathway has been shown to inhibit fibroblast activation, thereby alleviating SiO_2_-induced silicosis^[6]^. These findings suggest that targeting fibroblasts represents a promising strategy. However, developing efficient drugs to prevent the fibroblast-to-myofibroblast transition remains an area requiring further exploration.

Currently, only two drugs, pirfenidone and nintedanib, have been approved by the US Food and Drug Administration (FDA) for the treatment of pulmonary fibrosis, including silicosis^[7]^. However, both agents are associated with notable side effects, including indigestion, nausea, and diarrhea^[8]^. As a non-pharmacological treatment, lung transplantation improves the quality of life and prolongs survival in patients with pulmonary fibrosis^[9]^. Nonetheless, its therapeutic potential is limited by surgical complexity, scarcity of donor organs, and suboptimal long-term outcomes^[10]^. These challenges highlight the urgent need for novel, effective, and safe therapeutic strategies for silicosis treatment.

In recent years, natural compounds have attracted increasing attention as potential treatments for silicosis-induced pulmonary fibrosis, because of their superior biosafety and multi-target modes of action^[11]^. For instance, tetrandrine has been experimentally demonstrated to slow the progression of pulmonary fibrosis by downregulating the mRNA levels of pro-inflammatory and pro-fibrotic cytokines^[12]^. Similarly, oridonin has been shown to exert therapeutic effects in a mouse model of silica-induced pulmonary fibrosis^[13]^. These findings underscore the significant potential of natural compounds in treating silicosis. In our previous study, we screened a library of 3343 natural compounds and ultimately selected four plant-derived candidates for further investigation based on their diverse targets and inhibitory effects on fibroblasts. Among these, homoharringtonine (HHT) was identified as a particularly promising candidate.

HHT, a compound extracted from the *Cephalotaxus* genus, has been widely used in treating acute myeloid leukemia (AML) because of its ability to inhibit proliferation and promote apoptosis^[14]^. Additionally, it inhibits cardiac fibroblast activation and may reverse cardiac fibrosis^[15]^. Beyond AML, HHT has demonstrated therapeutic potential in treating liver cancer, breast cancer, and familial platelet disorder, among others^[16–18]^. For example, it has been shown to inhibit lung cancer cell viability and suppress the growth of lung tumors in mice^[19]^. Despite its broad therapeutic potential, no studies have yet explored HHT's efficacy in pulmonary fibrosis. Based on its known biological properties, we hypothesized that HHT could serve as a potential treatment for silicosis by inhibiting fibroblast activation, proliferation, and migration.

In the present study, we explored the effects of HHT on SiO_2_-induced pulmonary fibrosis in mice, as well as the underlying mechanisms, to validate the potential of HHT as a targeted therapeutic agent for silicosis.

## Materials and methods

### Preventive model of silicosis in mice

Male C57BL/6 mice aged six weeks were randomly divided into four groups (eight mice in each group): a saline control group, a silica group (50 mg/kg silica suspension), a silica + dimethyl sulfoxide (DMSO) control group, and a silica + HHT (1 or 2 mg/kg) group. Silica particles (size distribution: 99% between 0.5 and 10 μm, 80% between 1 and 5 μm, average particle diameter of 1.7 μm; Sigma-Aldrich, St. Louis, MO, USA) were suspended in 0.05 mL sterile normal saline and administered *via* a single intratracheal injection. The concentration of silica dust was selected based on previous experiments^[20]^. On the day the model was established, HHT (purity 99.75%; MedChemExpress, Princeton, NJ, USA) or vehicle was injected intraperitoneally every two days. After 28 days, the mice were sacrificed, and the lung tissues were collected and preserved at −80 ℃ for further study.

All procedures were conducted in strict accordance with the ethical principles of animal use and care, and were approved by the Institutional Animal Care and Use Committee of Nanjing Medical University on September 28, 2023 (Approval No. IACUC-2310011).

### Therapeutic model of silicosis in mice

Male C57BL/6 mice aged six weeks were randomly divided into five groups (eight mice in each group): the saline group, the silica group (50 mg/kg silica suspension), the silica + DMSO control group, the silica + rapamycin (2 mg/kg) group, and the silica + HHT (2 mg/kg) group. Silicosis in mice was induced by a single intratracheal injection of silica dust on day 0. Starting on day 28, rapamycin (MedChemExpress) and HHT were administered *via* intraperitoneal injection every two days at a dose of 2 mg/kg. The mice were sacrificed on day 56, and the lung tissues were harvested and preserved at −80 ℃ for further study.

### Histopathology analysis

Mouse lung tissues were fixed in 4% paraformaldehyde and embedded in paraffin. The tissues were sectioned using a microtome (Thermo Fisher ESC 350, Waltham, MA, USA) and dewaxed with xylene and ethanol. For hematoxylin and eosin (H&E) staining, the sections were stained with hematoxylin for 15 min, soaked in hydrochloric acid and ethanol for 30 s, and then stained with eosin for 2 min after washing. For Masson staining, the sections were counterstained using hematoxylin, ponceau, and toluidine blue.

We used a fibrosis scoring system to evaluate the pathological changes in lung tissues. Six mice per group were used for fibrosis scoring; the higher the score, the greater the degree of inflammation and fibrosis in lung tissues. The severity of the lesions was graded as follows: 0, zero/none; 1, marginal; 2, mild; 3, moderate; 4, severe; 5, very severe. The distribution of lesions was categorized as follows: 0, absent; 1, rare/occasional (10% of lung area); 2, sparse/limited (10%–25% of lung area); 3, moderate (25%–50% of lung area); 4, extensive/extensive (50%–75% of lung area); 5, very extensive/major (more than 75% of lung area). The fibrosis score in the control group was considered 0.

### Hydroxyproline content assay

We performed a hydroxyproline content assay (Cat. #A030-2, Jiancheng Bioengineering Institute, Nanjing, China) to detect the collagen content in mouse lung tissues. The sample to be tested was hydrolyzed with acid; the hydrolyzed solution was neutralized and filtered to obtain the test sample. We added 50 µL of the sample and 50 µL of chloramine T solution to a test tube or 96-well plate, mixed them well, and incubated the mixture at room temperature for 20 min. We then added 100 µL of DMAB solution (1 mol/L), mixed it well, and incubated it in a water bath at 60 ℃ for 15 min. Finally, we measured the absorbance at 560 nm using a spectrophotometer (LC-20AT/RF-10AXL, Shimadzu Corporation, Japan). A standard curve was generated to calculate the content of hydroxyproline in the samples.

### Hepatic, renal, and cardiac toxicity analysis

Alanine aminotransferase (ALT), aspartate aminotransferase (AST), blood urea nitrogen (BUN), serum creatinine (Scr), and mouse creatine kinase MB isoenzyme (CK-MB) were detected using enzyme-linked immunosorbent assay (ELISA) kits (Shanghai Hengyuan Biotechnology Co., Ltd., Shanghai, China) according to the manufacturer's instructions. Briefly, samples and standards were prepared, and biotinylated antigen was added, followed by incubation at 37 ℃ for 30 min. After washing, avidin-HRP was added and incubated at 37 ℃ for 30 min. After another wash, chromogenic solutions A and B were added, and the mixture was incubated at 37 ℃ for 10 min. The reaction was terminated by adding stop solution, and the optical density values were measured to calculate the concentrations of the target analytes.

### Cell culture and treatment

National Institutes of Health 3-day transfer mouse embryonic fibroblasts (NIH-3T3), human embryonic lung fibroblasts (MRC-5), and human monocytic leukemia cells (THP-1) were obtained from the American Type Culture Collection (ATCC, Manassas, VA, USA). NIH-3T3 and MRC-5 cells were cultured in Minimum Essential Medium (MEM; Life Technologies/Gibco, Grand Island, NY, USA), while THP-1 cells were cultured in RPMI Medium 1640 (RPMI 1640; Life Technologies/Gibco). All cultures were supplemented with 10% fetal bovine serum (FBS; Cat. #BISH1475, Biological Industries, Cromwell, CT, USA) and antibiotics (penicillin and streptomycin, Life Technologies/Gibco). Cells were maintained in a humidified incubator at 37 ℃ and 5% CO_2_.

THP-1 cells were differentiated into macrophages by incubation with phorbol 12-myristate 13-acetate (PMA) for 36 h. Differentiated macrophages were subsequently treated with 200 μg/mL SiO_2_ and 1 μmol/L HHT for 24 h. MRC-5 cells were incubated with TGF-β1 (Sigma-Aldrich) and HHT for 24 h. HHT was dissolved in DMSO and stored at −20 ℃.

### Western blotting

Total protein was extracted from cells and tissues using RIPA lysis buffer supplemented with phenylmethylsulfonyl fluoride (PMSF, SenBeiJia Biological Technology Co., Ltd., Nanjing, Jiangsu, China). For mouse lung tissues, the supernatant was collected after ultrasonic homogenization. The protein was quantified by the BCA protein quantification method and then mixed proportionally and stored at −20 ℃. For electrophoresis, the protein samples were mixed with sodium dodecyl sulfate (SDS) sample buffer (20%) and incubated on ice for 30 min. The samples were then boiled for 5 min, separated by 10% SDS-PAGE, and transferred onto a polyvinylidene fluoride (PVDF) membrane. The membrane was blocked in Tris-buffered saline containing 0.1% Tween-20 (TBST) and 5% skimmed milk at room temperature for 2 h, and then incubated with the primary antibody at 4 ℃ overnight. After washing with TBST for 15 min, the membrane was incubated with the corresponding secondary antibody at room temperature for 2 h. Detection was conducted using the ChemiDoc XRS+ imaging system (Bio-Rad Laboratories, Hercules, California, USA). Primary antibodies used for Western blotting are listed in ***Supplementary Table 1*** (available online).

### Quantitative reverse transcription-PCR (RT-qPCR)

Total RNA from cells was extracted using TRNzol Universal Reagent (TIANGEN, Beijing, China). The PARIS Kit Protein and RNA Isolation System (Invitrogen, Carlsbad, CA, USA) was used to isolate cytoplasmic and nuclear fractions of cells. A total of 500 ng RNA was reverse-transcribed using HiScript Ⅱ Q Select RT Supermix (Vazyme, Nanjing, China) or HiScript Ⅱ Q RT SuperMix for qPCR Kit (Vazyme) into complementary DNA (cDNA). SYBR Green Master Mix (Vazyme) and a real-time PCR system (Roche LightCycler 480 Ⅱ System, Basel, Switzerland) were used to quantify RNA expression. For conventional RT-PCR analysis, the 2× Taq plus Master Mix (Cat. #P112-01, Vazyme) was used to amplify cDNA. PCR products were separated on a 2% agarose gel pre-stained with ethidium bromide. The Gel Doc XR+ gel system (Bio-Rad Laboratories) was used for imaging. *GAPDH* was used as an internal control for mRNA. Primers used for RT-qPCR are listed in ***Supplementary Table 2*** (available online).

### Immunofluorescence staining

Samples were fixed with formaldehyde for 1 h and then washed with phosphate-buffered saline (PBS). Subsequently, they were blocked in 10% goat serum at room temperature for 1 h. The samples were then incubated with the primary antibody at 4 ℃ overnight. After washing with PBS, the secondary antibody (Cat. #A0516, Beyotime, Shanghai, China) was applied and incubated in the dark for 1 h. After washing, the samples were stained with DAPI (Cat. #C1005, Beyotime) for 15 min. Finally, an anti-fade mounting medium was applied, and the samples were observed and imaged under a microscope (LSM700B, Zeiss, Oberkochen, BW, Germany).

### EdU assays

Proliferation of MRC-5 cells was assessed using the Cell-Light EdU DNA Cell Proliferation Kit (RiboBio, Guangzhou, China). Briefly, cells seeded in 96-well plates were incubated with 100 μL of EdU medium at 37 ℃ with 5% CO_2_ for 2 h. After washing with PBS, cells were fixed with 4% formaldehyde for 30 min. Subsequently, cells were incubated with Apollo staining solution at room temperature in the dark for 30 min. Following the removal of the staining solution and a wash with permeabilization buffer, the cell nuclei were stained using 100 μL of 1× Hoechst 33342 solution per well for 30 min. After discarding the staining solution and washing with PBS, cells were observed and imaged under a fluorescence microscope (LSM700B, Zeiss).

### Wound-healing assay

The treated cells were seeded into 6-well plates and starved in medium containing 1% FBS for 12 h. When cells reached 90%–100% confluence, the cell layer was scratched with a sterilized 20 μL pipette tip. Images at multiple scratch points were captured immediately after scratching (0 h). The cells were then treated accordingly and cultured for 24 h. Scratch closure was observed and photographed under a microscope (LSM700B, Zeiss).

### Transwell migration assay

MRC-5 cells were seeded in the upper chamber of a 6-well Transwell insert (Costar, Cambridge, MA, USA). The corresponding treatments were added, and the cells were cultured for 24 h. The cells were fixed with 4% paraformaldehyde and stained with crystal violet. Non-migrated cells were removed using a cotton swab. The migrated cells were then viewed and photographed under a microscope (LSM700B, Zeiss).

### Ubiquitination assay

MRC-5 cells were treated with 20 μmol/L MG132 for 8 h before harvesting. Cells were lysed on ice in lysis buffer (1× PBS, 1% NP-40, 0.5% sodium deoxycholate, 1% SDS, 10 mmol/L *N*-ethylmaleimide, 1 mmol/L phenylmethylsulfonyl fluoride, 1 mmol/L Na_3_VO_4_, and 1 mmol/L NaF) for 10 min. Cell lysates were then diluted ten-fold with lysis buffer without SDS and subjected to immunoprecipitation using an ubiquitination antibody (Cat. #sc-8017, Santa Cruz Biotechnology, Dallas, TX, USA). The ubiquitination level of endogenous CCR1 was measured by Western blotting.

### CCK-8 cell viability assay

After transfection, cells (1 × 10^3^ cells/well) were seeded into a 96-well plate in triplicate. Cells were incubated for 1, 2, 3, and 4 days. Then the cells were treated with 100 μL of CCK-8 solution, and further incubated at 37 ℃ for 2 h. The absorbance was measured at 450 nm with a microtiter plate reader.

### Trypan blue staining

Cells were seeded in 6-well plates, treated according to experimental groups, and stained using a 0.4% trypan blue solution. The number of dead and living cells was observed under a microscope, and the survival rate of cells was calculated.

### Propidium iodide (PI) staining

We centrifuged the cell suspension at 2000 rpm for 5 min, resuspended the cells with PBS, adjusted the cell concentration to approximately 10^6^/mL, and added 5 μL of PI staining solution to 500 μL of cell suspension. The mixture was incubated at 4 ℃ in the dark for 30 min, followed by flow cytometry.

### Collagen gel contraction assay

According to the protocol for the rat tail tendon collagen type Ⅰ (Cat. #200110, Xinyou Biotechnology Co., Hangzhou, China), a digestive solution was used to convert the cells in the dish into a cell suspension, and then the cell suspension (5 × 10^5^) was placed in an ice bath. Next, 200 μL of mouse tail collagen type Ⅰ was added to 12 μL of 0.1 mol/L NaOH and mixed evenly. Subsequently, 23 μL of 10× PBS was added and mixed thoroughly, followed by 760 μL of the cell suspension. The final mixture was mixed well and transferred into a 12-well plate. The plate was incubated at room temperature for 20 min. After the gel solidified, cell culture medium was added to the wells, and the gel changes were recorded at different time points.

### Thermal proteome profiling

After suspension, cells were centrifuged at 1000 *g* for 5 min, and washed with PBS. The pellet was lysed using lysis buffer. Protein concentration was measured, and the samples were stored in cryopreservation solution at −80 ℃ until use. The proteins were digested into peptides and analyzed by liquid chromatography-tandem mass spectrometry, followed by bioinformatics analysis of the obtained data (Guangke Ander Biotechnology Co., Ltd., Hangzhou, China).

**Statistical analysis**

Student's *t*-test was used to compare differences between two groups, while analysis of variance (ANOVA) was used for comparisons among multiple groups. Each experiment was performed in triplicate, and statistical significance was defined as *P* < 0.05. Data analysis was performed using SPSS 26.0, and graphs were generated using GraphPad Prism 9.

## Results

### Candidate compounds were selected from the natural product library

We screened 4547 natural compounds from MedChemExpress's natural compound product library. Based on the criteria of maximizing compound diversity and target coverage, we initially narrowed the list to 532. Next, we treated MRC-5 cells with these compounds at a concentration of 10 μmol/L for 24 h, analyzed cell viability by the CCK-8 assay, and selected compounds that inhibited MRC-5 viability by more than 15% (*i.e.*, viability reduced to < 85%), ultimately reducing the number of target compounds to 29 (***Supplementary Fig. 1A***, available online).

Subsequently, MRC-5 cells were exposed to a concentration gradient of these compounds (0, 0.1, 1.0, and 5.0 μmol/L) for 24 h. The lowest concentration that reduced cell viability by more than 50% in the CCK-8 assay was defined as the optimal concentration for each compound (***Supplementary Fig. 1B***, available online). Additionally, pseudoprotodioscin exhibited no significant inhibitory effect even at a concentration of 10 μmol/L (***Supplementary Fig. 1C***, available online). Based on these findings, we excluded pseudoprotodioscin from further screening.

We performed trypan blue staining to assess the cytotoxicity of the remaining 28 compounds, and found that 18 compounds exhibited significant cytotoxic effects at their optimal concentrations (***Supplementary Fig. 2***, available online). We then performed PI staining on the remaining 10 compounds, and found that two compounds significantly increased the proportion of PI-positive cells (***Supplementary Fig. 3A***–***3C***, available online). These two compounds were therefore eliminated. Finally, eight compounds were identified through the screening process (four of which are plant-derived), including HHT. The CCK-8 assays demonstrated that treatment with HHT significantly inhibited the growth of MRC-5 cells in a concentration-dependent manner, with an IC_50_ of 88.75 μmol/L (***Supplementary Fig. 3D*** and ***3E***, available online).

### HHT blocked pulmonary fibrosis progression induced by SiO_2_

**Figure 1:**
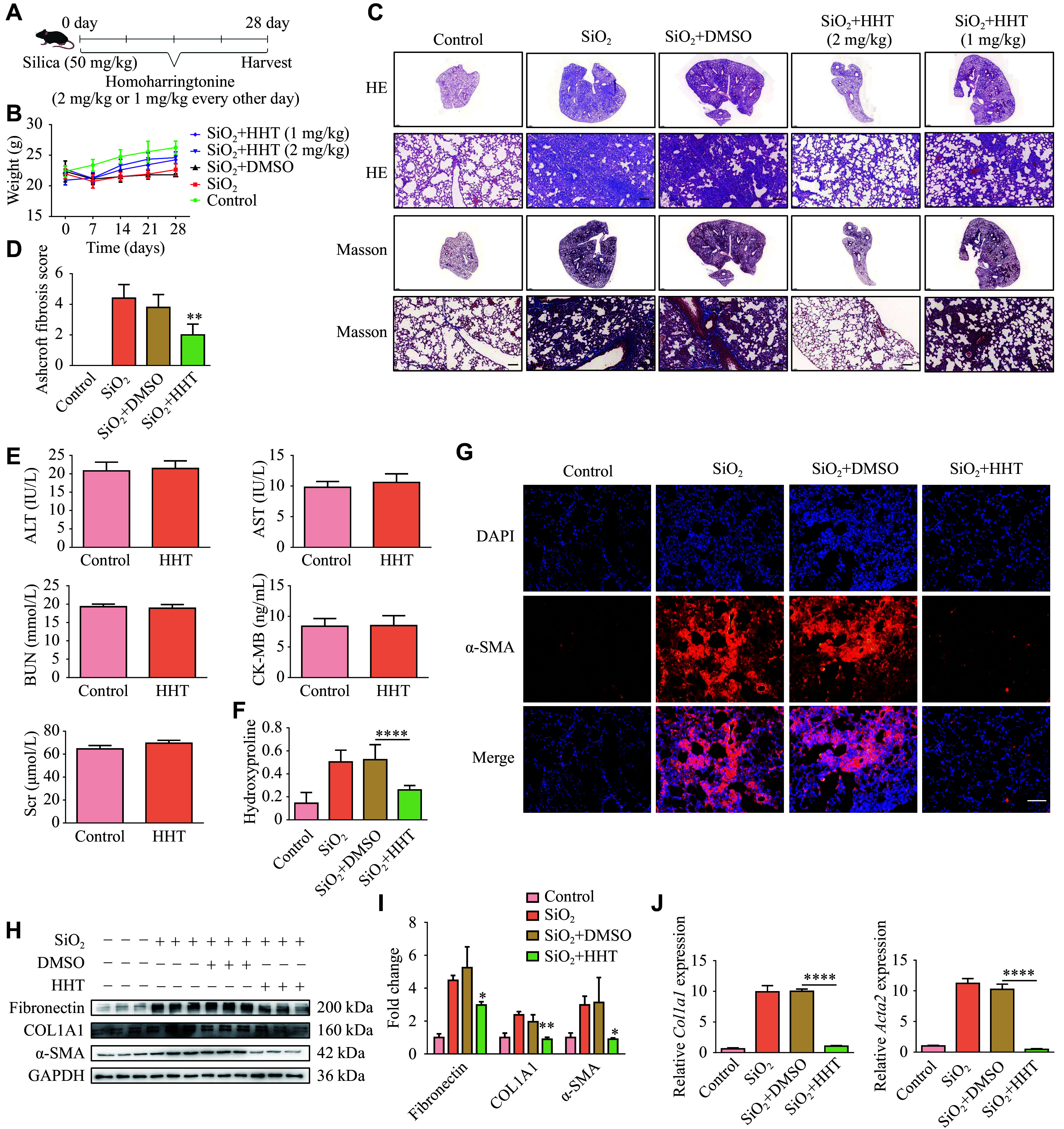
Homoharringtonine (HHT) inhibited SiO_2_-induced pulmonary fibrosis in mice. A: Schematic diagram of the HHT intervention model in silicosis mice with pulmonary fibrosis. B: Weight changes of the mice were recorded once a week. C: Results of hematoxylin and eosin and Masson staining in mouse lung tissues (magnification, 2× [upper] and 20× [lower]; scale bar, 100 μm). D: The fibrosis score of each group (*n* = 5). E: The changes in hepatorenal toxicity indices among mice in each group. F: The difference in hydroxyproline content among groups by enzyme-linked immunosorbent assay (*n* = 6). G: α-SMA expression in lung tissues of mice by immunohistochemistry (scale bar, 50 μm). H: Western blotting results of protein levels of fibronectin, COL1A1, and α-SMA in each group. I: Quantification of panel H. J: mRNA levels of *Col1a1* and *Acta2* in mouse lung tissues detected by quantitative reverse transcription-PCR (*n* = 4). Data are presented as mean ± standard deviation. Statistical analyses were performed using two-way ANOVA with Bonferroni's post hoc test. ^*^P < 0.05, ^**^P < 0.01, and^ ****^P < 0.0001 *vs.* the SiO_2_ + DMSO group. Abbreviations: Acta2, actin alpha 2; ALT, alanine aminotransferase; AST, aspartate aminotransferase; BUN, blood urea nitrogen; CK-MB, creatine kinase MB isoenzyme; COL1A1, collagen type Ⅰ alpha 1 chain; DMSO, dimethyl sulfoxide; Scr, serum creatinine; α-SMA, alpha smooth muscle actin; HHT, homoharringtonine; SiO_2_, silicon dioxide.

The mice received a single intratracheal injection of SiO_2_. Starting from the second day after modeling, HHT was administered every two days at a concentration of 1 mg/kg or 2 mg/kg. The mice were sacrificed on day 28 (***Fig. 1A***). Body weight was recorded weekly, and no significant changes were observed (***Fig. 1B***). H&E staining showed that fibrotic nodules in lung tissues were significantly reduced in the HHT treatment group compared with the SiO_2_-treated group. Similarly, Masson staining revealed that the HHT treatment significantly decreased SiO_2_-induced collagen deposition in mouse lung tissues (***Fig. 1C***). Moreover, both H&E and Masson staining showed that the effect of HHT at 2 mg/kg was more significant than that at 1 mg/kg. Therefore, we selected the 2 mg/kg concentration for the subsequent experiments. Additionally, the Ashcroft fibrosis score of the HHT-treated group was significantly lower than that in the SiO_2_-treated group (***Fig. 1D***).

We also examined the *in vivo* toxicity of HHT by measuring indicators of liver function (ALT, AST), kidney function (BUN, Scr), and heart function (CK-MB; ***Fig. 1E***). The results showed no significant difference between the HHT-treated group and the control group, indicating that HHT had no significant adverse effect *in vivo*. Additionally, HHT treatment significantly reduced the elevated hydroxyproline content in lung tissues of SiO_2_-treated mice (***Fig. 1F***). Immunofluorescence staining revealed that α-SMA levels were significantly increased in SiO_2_-treated mice but significantly decreased by HHT treatment (***Fig. 1G***). Western blotting showed that fibrosis-related markers (fibronectin, collagen Ⅰ, and α-SMA) were all significantly lower in the HHT-treated group than in the SiO_2_-treated group, indicating a reduction in pulmonary fibrosis by HHT (***Fig. 1H*** and ***1I***). Consistent results were observed in RT-qPCR assays, which showed a similar reduction in the expression levels of fibrosis-related genes (***Fig. 1J***). These results indicate that HHT intervention effectively attenuates SiO_2_-induced pulmonary fibrosis in mice.

### HHT inhibited TGF-β1-induced lung fibroblast activation *in vitro*

**Figure 2:**
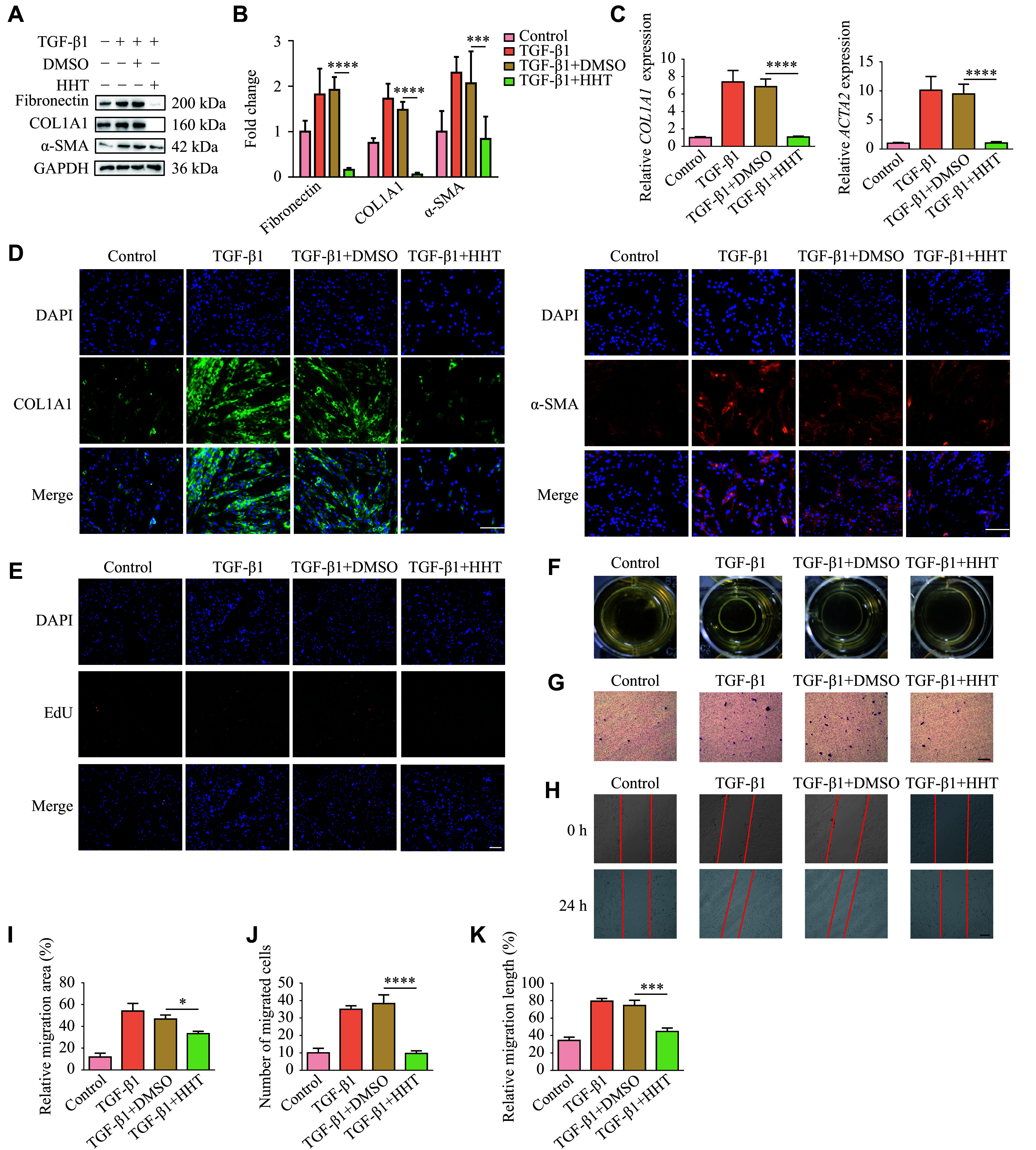
Homoharringtonine (HHT) inhibited TGF-β1-induced fibrosis and cell migration *in vitro.* MRC-5 cells were treated with HHT and TGF-β1 at a concentration of 1 μmol/L for 24 h simultaneously. A: Protein levels of fibronectin, collagen Ⅰ, and α-SMA in MRC-5 cells treated with TGF-β1 and HHT. B: Quantification of panel A. C: mRNA levels of *COL1A1* and *ACTA2* in MRC-5 cells treated with TGF-β1 and HHT, detected by quantitative reverse transcription-PCR (*n* = 4). D: Immunofluorescence staining of collagen Ⅰ and α-SMA in MRC-5 cells from different groups (scale bar, 100 μm). E: The proliferation of MRC-5 cells was detected by the EdU fluorescence staining (scale bar, 100 μm). F: Changes in collagen contraction among 3T3 cell groups were detected by a collagen gel contraction experiment. G: The migration ability of MRC-5 cells was detected by Transwell migration assay (scale bar, 100 μm). H: The migration ability of MRC-5 cells was detected by a wound-healing assay (scale bar, 100 μm). I: Quantification of panel F (*n* = 3). J: Quantification of panel G (*n* = 3). K: Quantification of panel H (*n* = 3). Data are presented as mean ± standard deviation. Statistical analyses were performed by two-way ANOVA with Bonferroni's post hoc test. ^*^P < 0.05, ^***^P < 0.001, and^ ****^P < 0.0001 *vs.* the TGF-β1 + DMSO group. Abbreviations: ACTA2, actin alpha 2; COL1A1, collagen type Ⅰ alpha 1 chain; DMSO, dimethyl sulfoxide; TGF-β1, transforming growth factor-β1; α-SMA, alpha smooth muscle actin.

We treated fibroblasts (MRC-5) with TGF-β1 to examine the inhibitory effect of HHT on fibroblast activation. MRC-5 cells were treated with HHT at a concentration of 1 μmol/L for 24 h. Western blotting showed that the protein levels of fibronectin, collagen, and α-SMA were significantly increased in the TGF-β1-treated group, compared with the control group, whereas HHT significantly reversed this trend (***Fig. 2A*** and ***2B***). Consistently, RT-qPCR assays revealed that HHT treatment significantly reduced the mRNA levels of *COL1A1* and *ACTA2* in TGF-β1-treated cells (***Fig. 2C***). We then performed immunofluorescence staining to detect the protein levels of fibrotic markers (collagen and α-SMA) in MRC-5 cells. The results showed that the fluorescence levels of these proteins in the HHT group were significantly lower than those in the TGF-β1 group, indicating the inhibitory effects of HHT on fibroblast activation (***Fig. 2D***). Additionally, EdU fluorescence staining demonstrated the ability of HHT to suppress cell proliferation (***Fig. 2E***). We next used 3T3, a mouse lung fibroblast cell line, for a mouse tail collagen gel contraction experiment, and found that HHT significantly inhibited the contraction ability of TGF-β1-treated 3T3 cells (***Fig. 2F*** and ***2I***). A Transwell migration assay showed that HHT significantly inhibited the migration of TGF-β1-treated MRC-5 cells (***Fig. 2G*** and ***2J***). Similarly, the wound-healing assay demonstrated a significant reduction in cell migration in the presence of HHT (***Fig. 2H*** and ***2K***). These results demonstrate the inhibitory effect of HHT on the activation, proliferation, and migration of TGF-β1-treated lung fibroblasts.

### CCR1 and PI3K/AKT signalings were potential targets of HHT in silicosis

**Figure 3:**
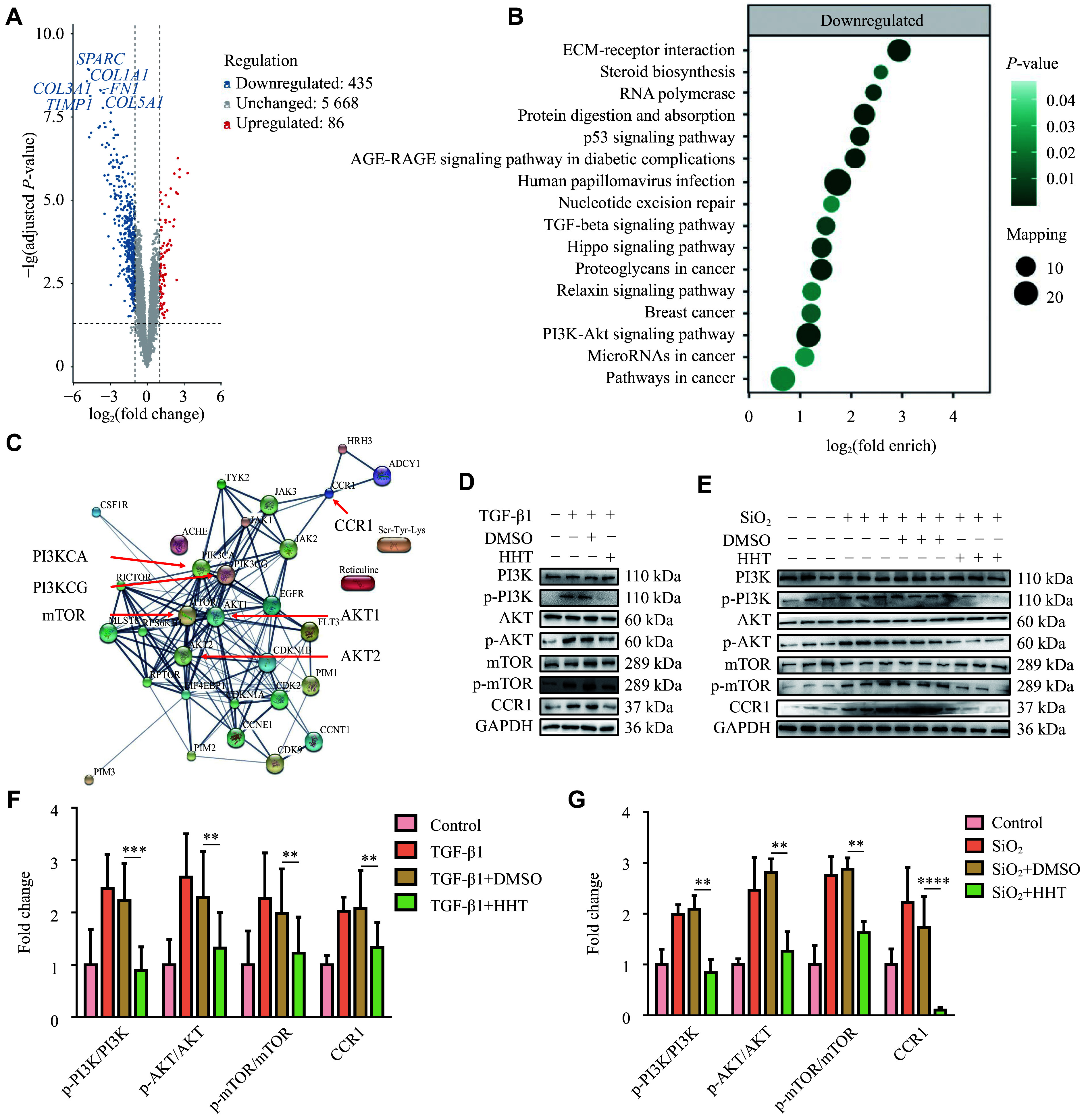
Exploring the mechanism of homoharringtonine (HHT) inhibiting pulmonary fibrosis. A: The volcano plot showing differentially expressed genes between the TGF-β1 and the TGF-β1 + HHT groups. Blue represents downregulated genes, and red represents upregulated genes. B: KEGG pathway enrichment analysis of differentially expressed proteins between the TGF-β1 and the TGF-β1 + HHT groups. C: PPI network analysis predicting the associations among the top 30 targets (http://string-db.org). D and E: Western blotting analysis of PI3K/AKT/mTOR signaling pathway-related molecules between groups *in vitro* (D) and *in vivo* (E) after HHT treatment. F: Quantification of panel D (*n* = 3). G: Quantification of panel E (*n* = 3). Data are presented as mean ± standard deviation. Statistical analyses were performed by two-way ANOVA with Bonferroni's post hoc test. ^**^P < 0.01, ^***^P < 0.001, and^ ****^P < 0.0001 *vs.* the TGF-β1 + DMSO group (F) or the SiO_2_ + DMSO group (G). Abbreviations: AKT, protein kinase B; CCR1, chemokine (C-C motif) receptor 1; DMSO, dimethyl sulfoxide; mTOR, mammalian target of rapamycin; TGF-β1, transforming growth factor-β; PI3K, phosphatidylinositol 3-kinase.

We identified and quantified a total of 6517 proteins using thermal proteome profiling with MRC-5 cells selected for the analysis. The volcano plot showed that fibrosis-related molecules COL1A1, COL3A1, COL5A1, and FN1 were significantly reduced after HHT treatment, indicating the potential of HHT in fibrosis modulation (***Fig. 3A*** and ***Supplementary Table 3*** [available online]). Through KEGG pathway analysis, we identified a canonical signaling pathway in fibrosis, the PI3K-AKT signaling pathway, and hypothesized that HHT may play a role through this signaling pathway (***Fig. 3B*** and ***Supplementary Fig. 3F*** [available online]).

To elucidate HHT's inhibitory mechanism on lung fibroblast activation, we performed target prediction using the SwissTargetPrediction platform (https://www.swisstargetprediction.ch) and identified potential targets associated with the inhibition of fibroblast activation by HHT (***Supplementary Table 4***, available online). Subsequent protein-protein interaction (PPI) analysis of the top 30 predicted targets revealed that the PI3K/AKT/mTOR signaling pathway was the most abundant pathway that interacted with other molecules (***Fig. 3C***), suggesting that HHT may exert its effects through this signaling pathway. Additionally, the chemokine receptor CCR1, which is well-known for its association with fibroblast migration in pulmonary fibrosis^[21]^, was also identified (***Fig. 3C***). Considering that our previous cell experiments had already verified the inhibitory potential of HHT on cell migration, we hypothesized that HHT might inhibit cell migration by modulating CCR1.

We further validated the above findings by Western blotting. In TGF-β1-treated MRC-5 cells, the protein levels of phosphorylated PI3K (p-PI3K), AKT (p-AKT), and mTOR (p-mTOR) were significantly reduced after HHT treatment, as well as those of CCR1 (***Fig. 3D*** and ***3F***). These results were further confirmed *in vivo*, as evidenced by HHT-mediated decreases in the activation of both PI3K/AKT/mTOR signaling and CCR1 in lung tissues of SiO_2_-treated mice (***Fig. 3E*** and ***3G***). These findings suggest that HHT may exert its inhibitory effects on SiO_2_-induced silicosis in mice through the CCR1 and PI3K/AKT signaling pathways.

### HHT inhibited fibroblast migration by inducing CCR1 protein ubiquitination

**Figure 4:**
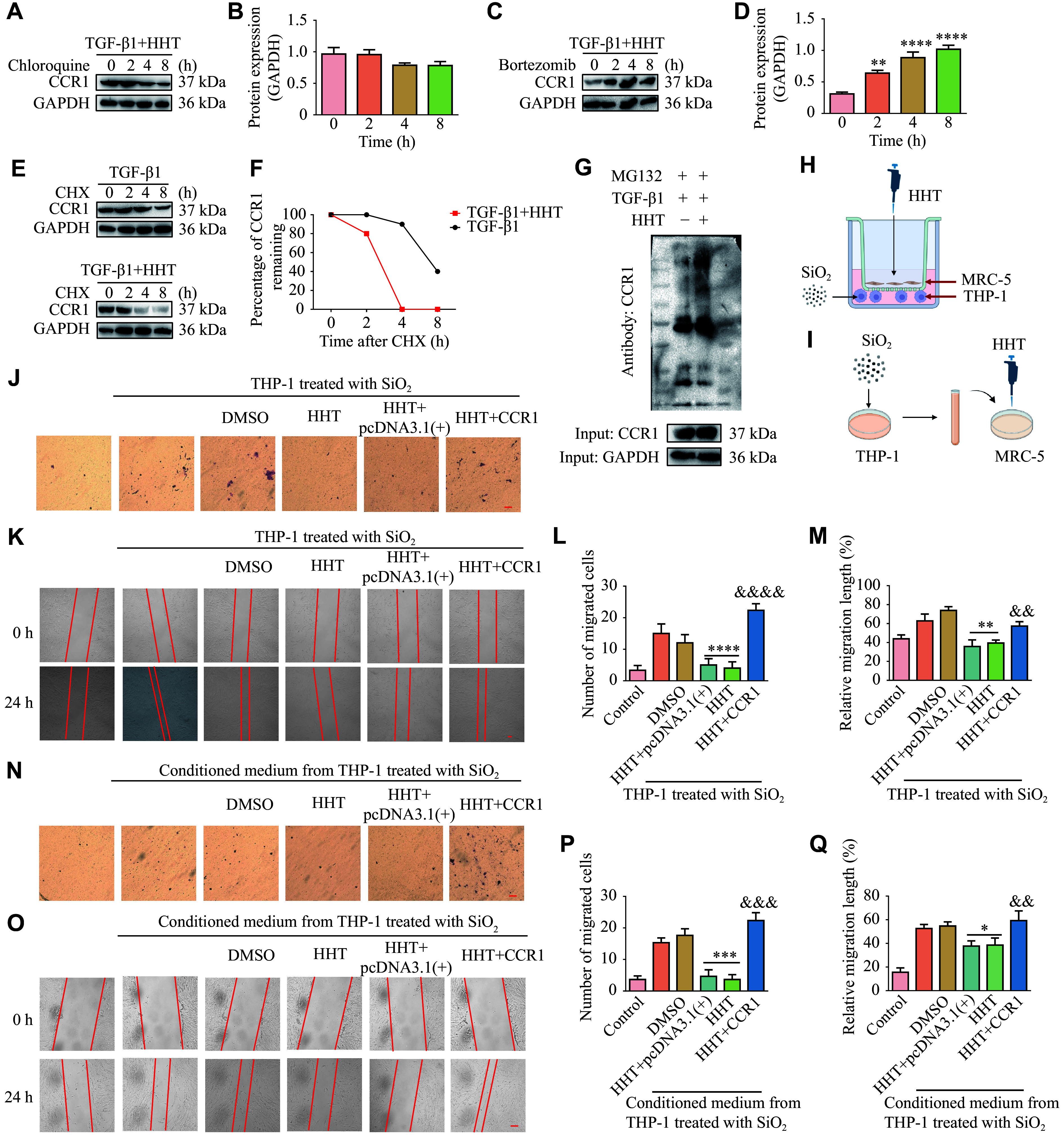
Homoharringtonine (HHT) inhibited fibroblast migration by affecting CCR1. A: MRC-5 cells were treated with TGF-β1 and HHT for 24 h, then treated with 20 μmol/L chloroquine for 0, 2, 4, and 8 h. B: Quantification of panel A (*n* = 3). C: MRC-5 cells were treated with TGF-β1 and HHT for 24 h, then treated with 100 nmol/L bortezomib for 0, 2, 4, and 8 h. D: Quantification of panel C (*n* = 3). E: MRC-5 cells were treated with TGF-β1 and HHT for 24 h, then treated with 20 μmol/L CHX for 0, 2, 4, and 8 h. F: Quantification of panel E (*n* = 3). G: Ubiquitination assay was performed to detect the ubiquitination levels of CCR1 in MRC-5 cells treated with HHT. H and I: Macrophage and fibroblast co-culture model. J–M: The migration ability of fibroblasts was detected by Transwell migration assay (J; scale bar, 100 μm) and wound-healing assay (K; scale bar, 100 μm). L and M: Quantification of panels J (L; *n* = 3) and K (M; *n* = 3). N–Q: The migration ability of fibroblasts was detected by Transwell migration assay (N; scale bar, 100 μm) and wound-healing assay (O; scale bar, 100 μm). Quantification of panels N (P; *n* = 3) and O (Q; *n* = 3). Data are presented as mean ± standard deviation. Statistical analyses were performed by two-way ANOVA with Bonferroni's post hoc test. ^*^P < 0.05, ^**^P < 0.01, ^***^P < 0.001, and ^****^P < 0.0001 *vs.* the 0 h group (D) or the SiO_2_ + DMSO group (L–Q); ^&&^*P* < 0.01, ^&&&^*P* < 0.001, and ^&&&&^*P* < 0.0001 *vs.* the SiO_2_ + HHT + pcDNA3.1(+) group. Abbreviations: DMSO, dimethyl sulfoxide; HHT, homoharringtonine.

To elucidate the underlying mechanism by which HHT mediates CCR1 downregulation, we treated MRC-5 cells with TGF-β1 and autophagy inhibitor chloroquine. The results showed that CCR1 protein levels were not affected by chloroquine (***Fig. 4A*** and ***4B***), indicating that HHT did not induce CCR1 downregulation through autophagy. Next, we treated MRC-5 cells with TGF-β1 and proteasome inhibitor bortezomib, and found that CCR1 protein levels significantly increased in a time-dependent manner (***Fig. 4C*** and ***4D***). Therefore, we hypothesized that HHT might affect the ubiquitination of CCR1. We treated MRC-5 cells with cycloheximide (CHX), a reagent commonly used for protein half-life detection because of its ability to inhibit protein synthesis, and found that CCR1 protein degradation was faster in the HHT treatment group than in the TGF-β1 treatment group (***Fig. 4E*** and ***4F***). We also found that the ubiquitination level of CCR1 significantly increased in the HHT treatment group, compared with the TGF-β1 treatment group (***Fig. 4G***). These results indicate that HHT induces CCR1 downregulation by promoting its ubiquitination.

Since CCR1 performs its role by binding to its ligands (such as CCL3, CCL5, and CCL7), which are mainly secreted by macrophages^[22]^, we constructed co-culture models using macrophages and lung fibroblasts. First, macrophages and fibroblasts were co-cultured using inserts, with SiO_2_-stimulated macrophages in the lower chamber and HHT-treated fibroblasts in the upper chamber (***Fig. 4H***). Additionally, we used the CCR1 overexpression plasmid to eliminate the effect of HHT treatment. The Transwell migration assay showed that, in the co-culture model, the number of migrated cells decreased significantly after overexpression of CCR1 (***Fig. 4J*** and ***4L***). Similarly, the wound-healing assay demonstrated a consistent reduction in cell migration (***Fig. 4K*** and ***4M***). In another model, we treated fibroblasts with the conditioned medium from SiO_2_-treated macrophages for 24 h (***Fig. 4I***). Both the Transwell migration assay and the wound-healing assay showed that HHT inhibited fibroblast migration by reducing the expression of CCR1 (***Fig. 4N***–***4Q***). These results indicate that HHT inhibits fibroblast migration by affecting the ubiquitination of CCR1.

### HHT inhibited pulmonary fibroblast activation by inhibiting the PI3K/AKT/mTOR phosphorylation

**Figure 5:**
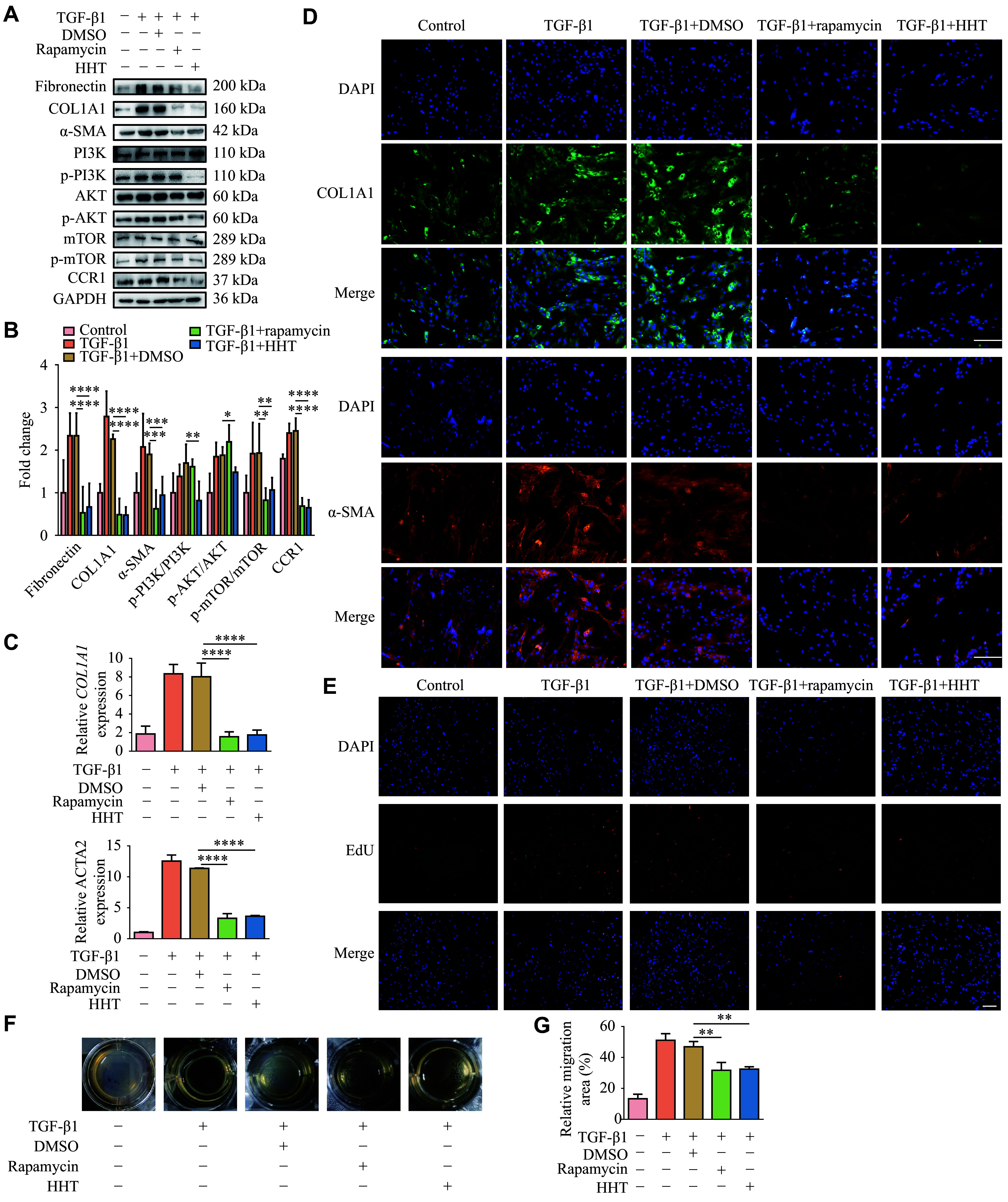
Homoharringtonine (HHT) inhibited pulmonary fibrosis by inhibiting the PI3K/AKT/mTOR pathway. Rapamycin (25 mmol/L) was co-administered with TGF-β1 and HHT for 24 h in MRC-5 cells. A: The protein levels of the PI3K/AKT/mTOR signaling pathway were compared between MRC-5 cell groups. B: Quantification of panel A (*n* = 3). C: Quantitative reverse transcription-PCR was used to detect the differences in mRNA levels of fibrosis markers between the MRC-5 cell groups (*n* = 4). D: The differences in fibrosis markers between the MRC-5 cell groups were detected by immunofluorescence staining (scale bar, 100 μm). E: The proliferation of MRC-5 cells was detected by the EdU fluorescence staining (scale bar, 100 μm). F: Changes in collagen contraction among 3T3 cell groups were detected by the collagen gel contraction experiment. G: Quantification of panel F (*n* = 3). Data are presented as mean ± standard deviation. Statistical analyses were performed by two-way ANOVA with Bonferroni's post hoc test. ^*^P < 0.05, ^**^P < 0.01, ^***^P < 0.001, and^ ****^P < 0.0001 *vs.* the TGF-β1 + DMSO group. Abbreviations: AKT, protein kinase B; DMSO, dimethyl sulfoxide; mTOR, mammalian target of rapamycin; PI3K, phosphatidylinositol 3-kinase; TGF-β1, transforming growth factor-β1.

To validate whether HHT inhibits fibroblast activation by affecting the PI3K/AKT/mTOR signaling pathway, we also used the mTOR inhibitor rapamycin to investigate the inhibitory effects of HHT in TGF-β1-activated fibroblasts (MRC-5). Western blotting showed that both HHT and rapamycin significantly decreased the protein levels of fibrosis markers and phosphorylated mTOR, while HHT also significantly decreased the protein levels of p-PI3K and p-AKT (***Fig. 5A*** and ***5B***). Notably, rapamycin also significantly downregulated CCR1 protein levels in these activated cells. RT-qPCR showed that both rapamycin and HHT reduced mRNA levels of *COL1A1* and *ACTA2* (***Fig. 5C***). The fluorescence intensity of collagen Ⅰ and α-SMA in the rapamycin and the HHT-treated groups was significantly reduced compared with the control group (***Fig. 5D***). The EdU fluorescence staining showed that the number of proliferating cells was significantly lower in the rapamycin and HHT treatment groups, compared with the control group (***Fig. 5E***). Similarly, the collagen contraction experiment also revealed the inhibitory effects of both on collagen contraction (***Fig. 5F*** and ***5G***). The PI3K activator 740Y-P reversed the inhibitory effects of HHT on TGF-β1-induced fibroblast activation and proliferation (***Supplementary Fig. 4***, available online). Collectively, these findings indicate that HHT inhibits fibroblast activation by blocking phosphorylation of the PI3K/AKT/mTOR signaling pathways *in vitro*.

### HHT showed promising therapeutic potential in the silicosis mouse model

**Figure 6:**
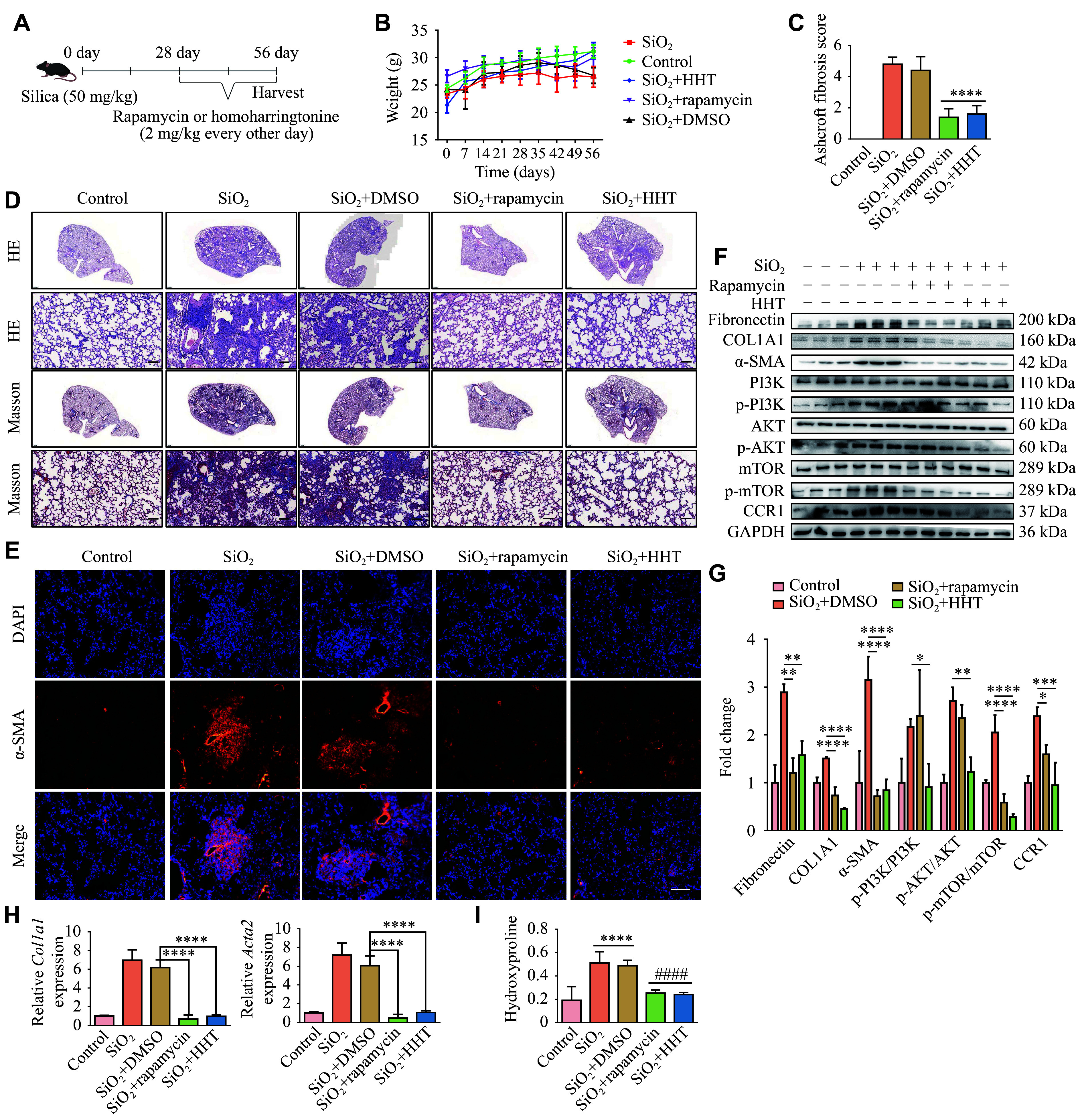
Homoharringtonine (HHT) inhibited SiO_2_-induced pulmonary fibrosis in mice by inhibiting the PI3K/AKT/mTOR pathway. A: Rapamycin and HHT treatment in the mouse model of pulmonary fibrosis. B: The body weight of the mice was recorded weekly. C: The fibrosis score of each group (*n* = 5). D: Hematoxylin and eosin and Masson staining results of mouse lung tissues in the rapamycin and HHT treatment groups (magnification, 2× [upper] and 20× [lower]; scale bar, 100 μm). E: α-SMA expression in mouse lung tissues detected by immunohistochemistry (scale bar, 50 μm). F: The protein levels of fibrosis markers and the PI3K/AKT/mTOR signaling pathway in mouse lung tissues were detected by Western blotting. G: Quantification of panel F (*n* = 3). H: The mRNA levels of pulmonary fibrosis markers in mouse lung tissues were detected by quantitative reverse transcription-PCR (*n* = 4). I: The content of hydroxyproline in mouse lung tissues was detected by enzyme-linked immunosorbent assay (*n* = 6). Statistical analyses were performed by two-way ANOVA with Bonferroni's post hoc test. ^*^P < 0.05, ^**^P < 0.01, ^***^P < 0.001, and^ ****^P < 0.0001 *vs.* the SiO_2_ + DMSO group; ^ ####^*P* < 0.0001 *vs.* the SiO_2_ + rapamycin group. Abbreviations: AKT, protein kinase B; CCR1, chemokine (C-C motif) receptor 1; DMSO, dimethyl sulfoxide; PI3K, phosphatidylinositol 3-kinase; mTOR, mammalian target of rapamycin.

Finally, we explored whether HHT has a therapeutic effect in the silicosis mouse model. The mice were given rapamycin or HHT at 2 mg/kg every two days starting on day 28 after SiO_2_ injection, and then the mice were sacrificed on day 56 (***Fig. 6A***). There were no significant differences in body weight changes among the groups (***Fig. 6B***). H&E and Masson staining revealed that both rapamycin and HHT significantly reduced the severity of pulmonary fibrosis in silicosis mice, demonstrating their therapeutic effects (***Fig. 6D***). The fibrosis scores of the rapamycin- and the HHT-treated groups were significantly lower than those of the SiO_2_-treated group (***Fig. 6C***). Additionally, the fluorescence intensity of α-SMA in lung tissues of mice treated with rapamycin or HHT was significantly reduced (***Fig. 6E***). Western blotting showed that the protein levels of fibronectin, collagen Ⅰ, and α-SMA, as well as phosphorylated mTOR, were significantly reduced in rapamycin-treated mice. Moreover, HHT treatment significantly reduced p-PI3K and p-AKT levels in SiO_2_-treated mice, compared with those in the rapamycin group (***Fig. 6F*** and ***6G***). Compared with the control group treated with SiO_2_, the mRNA levels of *Col1a1* and *Acta2* were significantly reduced in both the rapamycin- and the HHT-treated groups (***Fig. 6H***). Finally, through the detection of hydroxyproline content, we demonstrated the therapeutic effects of rapamycin and HHT on SiO_2_-induced pulmonary fibrosis in mice (***Fig. 6I***).

## Discussion

**Figure 7:**
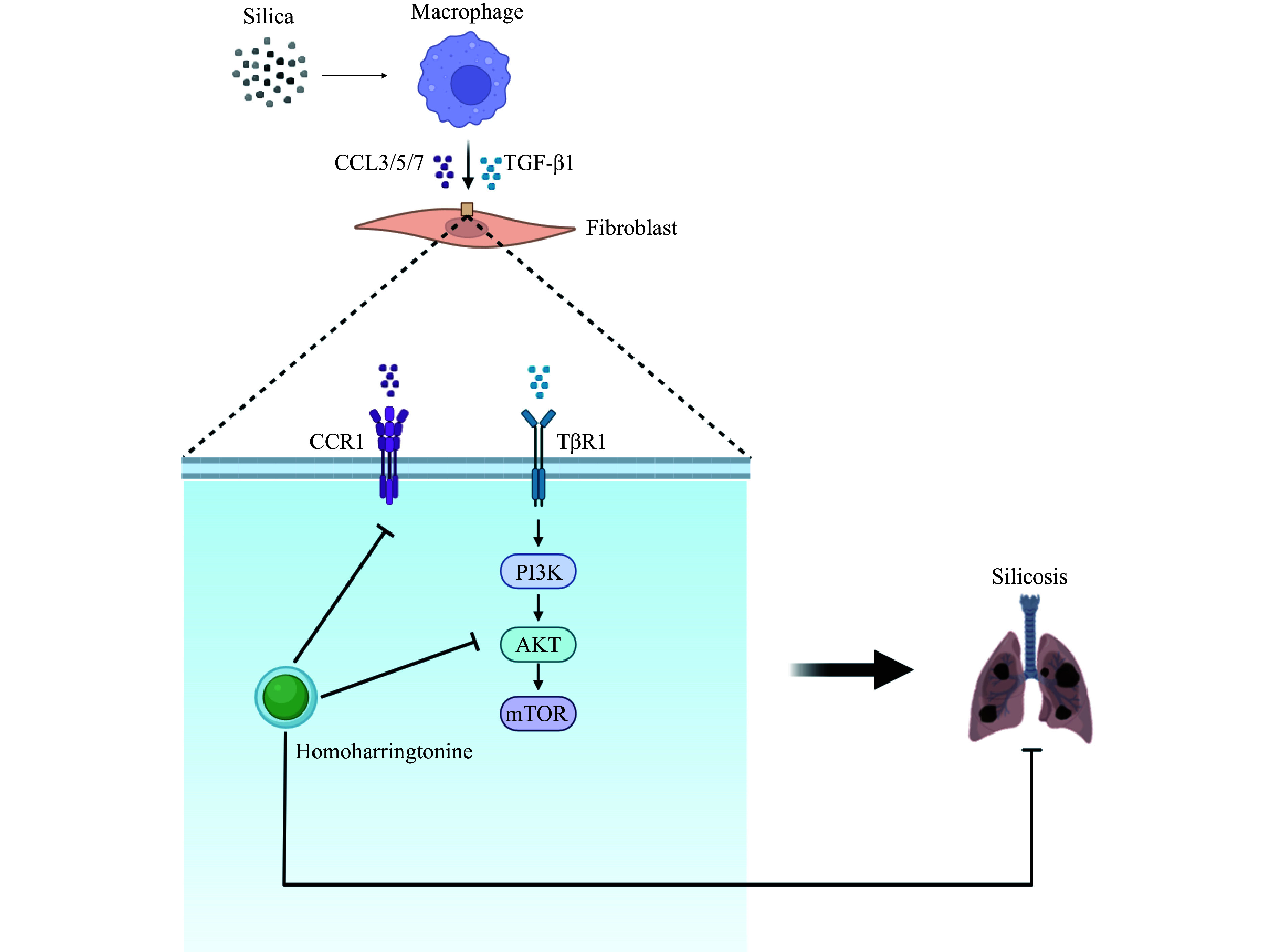
Diagram of homoharringtonine (HHT)-related anti-silicosis mechanism. SiO_2_ stimulates and activates macrophages, which secrete cytokines CCL3/5/7 and TGF-β1. On one hand, CCL3/5/7 binds to CCR1 on the lung fibroblast membrane to promote cell migration. On the other hand, TGF-β1 promotes fibroblast activation through the PI3K/AKT/mTOR signaling pathway. HHT exerts its anti-silicosis effect by inhibiting both the CCR1 and PI3K/AKT/mTOR signaling pathways. Abbreviations: CCL3/5/7, C-C motif chemokine ligands 3, 5, and 7; TGF-β1, transforming growth factor-beta 1; CCR1, C-C motif chemokine receptor 1; TβR1, TGF-beta receptor 1; PI3K, phosphatidylinositol 3-kinase; mTOR, mammalian target of rapamycin.

Through natural compound-based drug screening, we identified HHT as a promising therapeutic candidate for pulmonary fibrosis that targets fibroblasts. The present study demonstrated that HHT inhibited pulmonary fibroblast activation by suppressing the PI3K/AKT/mTOR signaling pathway and reduced fibroblast migration by promoting the ubiquitination and degradation of CCR1. Additionally, HHT exerted both therapeutic and interventional effects on SiO_2_-induced pulmonary fibrosis in mice. As a natural compound, HHT offers a more favorable safety profile compared with traditional drugs, making it a promising candidate for the treatment of silicosis (***Fig. 7***).

To identify new drugs for silicosis treatment, we focused our research on natural compounds. From a library of 3343 natural compounds, we screened for the optimal concentration that inhibits fibroblast proliferation, excluded those with significant cytotoxicity, and obtained eight compounds. Given their broader availability and lower toxicity, we prioritized four plant-derived natural compounds: HHT, tomatine, brusatol, and γ-mangostin. Notably, previous studies have shown that HHT exhibits a strong inhibitory effect on cardiac fibroblasts, which is consistent with our findings^[15]^. As there are currently no studies investigating the effects of HHT in pulmonary fibrosis, we selected it as the focus of this study, aiming to explore its potential as a novel candidate drug for silicosis treatment.

HHT, an alkaloid derived from *Cephalotaxus* species, has been extensively studied for its potent antitumor activity and favorable extraction yield. Its anti-tumor effects are attributed to the blockade of nascent peptide chain elongation and subsequent inhibition of protein synthesis^[23]^, with demonstrated efficacy in treating various types of cancer^[24]^. Despite its widespread use in oncology, there is limited research on the role of HHT in fibrosis-related diseases. One study demonstrated that HHT inhibited knee arthrofibrosis through the PI3K signaling pathway, and also revealed its inhibitory effect on fibroblast proliferation^[25]^. In our silicotic model, silicotic nodules formed on day 28, and complete silicotic nodules were observed on day 56. Therefore, we administered HHT to mice at different time points to evaluate its intervention and treatment effects on SiO_2_-induced pulmonary fibrosis. Additionally, we evaluated its biosafety by assessing hepatorenal and cardiac toxicity, and found that HHT had a favorable safety profile. Our findings suggest that HHT interferes with silicosis by inhibiting fibroblast activation and migration, offering potential as a novel therapeutic option.

Fibroblasts are critical effector cells in the pathogenesis of pulmonary fibrosis. Upon activation by various cytokines and signaling pathways, fibroblasts proliferate, differentiate, and eventually transform into myofibroblasts^[26]^. Myofibroblasts are considered the primary effector cells in silicosis-associated fibrosis, characterized by enhanced contractility, increased secretion of collagen and extracellular matrix, and elevated α-SMA expression. Preventing fibroblast activation or promoting myofibroblast apoptosis is essential for resolving fibrosis^[27]^. Additionally, targeting the migratory capacity of lung fibroblasts may also be a potential therapeutic strategy for pulmonary fibrosis^[28]^. In the present study, we observed that HHT significantly inhibited proliferation, activation, and migration in TGF-β1-activated lung fibroblasts (MRC-5). These findings further support the anti-fibrotic potential of HHT in silicosis.

To identify the pathway through which HHT exerts its anti-fibrotic potential, we predicted the protein-binding targets of HHT and performed PPI analysis on the top 30 potential targets. PPI analysis identified the PI3K/AKT/mTOR signaling axis as the most prominently involved pathway in HHT's anti-fibrotic effects in silicosis, consistent with the findings from KEGG analysis. This pathway plays diverse roles in regulating cell growth, proliferation, apoptosis, migration, metabolism, and survival^[29]^. It is also pivotal in driving the expression of pro-inflammatory mediators and recruiting inflammatory cells, contributing significantly to the progression of lung inflammation and fibrosis. mTOR acts as a regulator that controls cell metabolism, growth, and proliferation, and its inhibitor, rapamycin, has demonstrated efficacy in reducing inflammation and fibrosis, such as in non-alcoholic steatohepatitis^[30]^. Based on these insights, we used rapamycin as a positive control in parallel with HHT to evaluate their effects. Both *in vivo* and *in vitro* experiments demonstrated that HHT inhibited the PI3K/AKT/mTOR pathway by suppressing PI3K/AKT/mTOR phosphorylation. Notably, both HHT and rapamycin effectively treated SiO_2_-induced pulmonary fibrosis in mice and alleviated fibrosis by inhibiting fibroblast activation and migration. Furthermore, HHT demonstrated greater therapeutic efficacy than rapamycin, likely due to its ability to act on multiple targets simultaneously. These findings highlight the potential of HHT as a superior treatment for silicosis.

Another molecule of interest in target prediction is CCR1. Chemokine receptors, including CCR1, are heterogeneous and can be activated by a range of chemokines. CCR1 is highly expressed in immune and inflammatory cells, transmitting signals through interactions with ligands, such as CCL3, CCL5, and CCL7. Notably, the binding of CCL3 to CCR1 has been shown to cause fibroblast aggregation in a bleomycin-induced pulmonary fibrosis model, ultimately leading to fibrosis^[21]^. Previous studies have demonstrated that CCR1 inhibitors significantly reduce lung inflammation and fibrosis^[31]^, aligning with our findings.

Our results demonstrated that CCR1 protein levels were reduced following HHT treatment. Since CCR1 is not regulated by phosphorylation, we hypothesized that HHT might promote its degradation. Subsequent experiments demonstrated that HHT increased CCR1 ubiquitination, particularly in the presence of TGF-β1. Furthermore, in co-culture experiments with silica-stimulated macrophages, HHT reduced the migration of fibroblasts by inhibiting the expression of CCR1. These findings suggest that HHT regulates CCR1 through ubiquitination, which contributes to its anti-fibrotic effects by inhibiting fibroblast migration and activation.

However, the present study has several limitations. First, we evaluated the interventional and therapeutic effects of HHT in a murine silicosis model only, without incorporating clinical samples, limiting the translational relevance of our findings. Second, although HHT is known for its multi-target activity, we focused primarily on the PI3K/AKT pathway and CCR1, leaving other potential targets unexplored. Third, we did not investigate the mechanistic relationship between CCR1 and the PI3K/AKT/mTOR signaling pathway, assessing their effects on fibroblasts independently. Future research should aim to clarify this connection to provide a more integrated understanding of HHT's mechanism of action. Lastly, while we studied the effects of HHT on human fibroblasts, its effect on other effector cells, such as epithelial and endothelial cells, or primary lung fibroblasts, was not assessed. Future studies are needed to explore the therapeutic potential of HHT across multiple cell types and signaling pathways involved in silicosis.

In conclusion, our findings demonstrate that HHT can effectively prevent and treat SiO_2_-induced pulmonary fibrosis in mice, primarily by inhibiting the PI3K/AKT/mTOR signaling pathway and CCR1 expression. By screening a natural compound library targeting fibroblast activity, we identified HHT and demonstrated its therapeutic potential for pulmonary fibrosis through both *in vivo* and *in vitro* experiments. These results provide novel insights into silicosis treatment strategies and highlight HHT as a promising candidate for further preclinical and clinical investigation.

## SUPPLEMENTARY DATA

Supplementary data to this article can be found online.
