# Supplementary material for: Homoharringtonine exerts anti-silicosis effects by inhibiting the CCR1 and PI3K/AKT signaling pathways in lung fibroblasts
Source: J Biomed Res. 2025 May 21;39(6):622–38. doi: 10.7555/JBR.39.20250074 (PMC12683513; doi:10.7555/JBR.39.20250074)
Supplement: Supplementary file 1 — Supplementary data to this article can be found online. [file jbr-39-6-622-Supplementary.pdf]

Available online at www.jbr-pub.org.cn

Open Access at PubMed Central

JBR

Journal of Biomedical Research, 2025 39(6): 1–7

Supplementary Data

Homoharringtonine exerts anti-silicosis effects by inhibiting the CCR1 and PI3K/AKT signaling pathways in lung fibroblasts

Xinying Jia[1], Ziwei Li[1], Xiyue Hu[2,3], Ting Wang[1,4], Wenxiu Lian[1], Wenqing Sun[5], Yi Liu[1,✉], Chunhui Ni[1,2]

[1]*Department of Occupational Medicine and Environmental Health, Key Laboratory of Modern Toxicology of Ministry of Education, School of Public Health, Nanjing Medical University, Nanjing, Jiangsu 211166, China;*
[2]*Department of Public Health, Kangda College of Nanjing Medical University, Lianyungang, Jiangsu 222000, China;*
[3]*Community Health Service Center of Anzhen Street, Xishan District, Wuxi, Jiangsu 214035, China;*
[4]*Department of Pathology, Nanjing Drum Tower Hospital, the Affiliated Hospital of Nanjing University Medical School, Nanjing, Jiangsu 210000, China;*
[5]*The Affiliated Wuxi Center for Disease Control and Prevention of Nanjing Medical University, Wuxi Center for Disease Control and Prevention, Wuxi Medical Center, Nanjing Medical University, Wuxi, Jiangsu 214400, China.*

Supplementary Table 1 **Antibodies used for Western blotting and immunofluorescence (IF) staining**

Antibodies	Dilution ratio
GAPDH (ABclonal, AC002)	1 ∶ 1 000 for WB
Fibronectin (Abcam, ab45688)	1 ∶ 1 000 for WB
COL1A1 (ABclonal, A1352)	1 ∶ 1 000 for WB; 1 ∶ 400 for IF
α-SMA (Abcam, ab32575)	1 ∶ 1 000 for WB; 1 ∶ 400 for IF
Phospho-AKT (ABclonal, AP1208)	1 ∶ 1 000 for WB
Phospho-mTOR (ABclonal, AP0978)	1 ∶ 1 000 for WB
Phospho-PI3K (Affinity, AF3242)	1 ∶ 1 000 for WB
AKT (HUABIO, ST48-09)	1 ∶ 1 000 for WB
Anti-mTOR (HUABIO, SU30-00)	1 ∶ 1 000 for WB
PI3K (ABclonal, A22730)	1 ∶ 1 000 for WB
CCR1 (ABclonal, A18341)	1 ∶ 1 000 for WB

✉Corresponding author: Chunhui Ni and Yi Liu, Department of Occupational Medicine and Environmental Health, Key Laboratory of Modern Toxicology of Ministry of Education, School of Public Health, Nanjing Medical University, Nanjing, Jiangsu 211166, China. E-mails: chninjmu@126.com (Ni) and liuyi323@njmu.edu.cn (Liu).

Received: 21 February 2025; Revised: 07 May 2025; Accepted: 18 May 2025; Published online: 21 May 2025

CLC number: R135.2, Document code: A

The authors reported no conflict of interests.

This is an open access article under the Creative Commons Attribution (CC BY 4.0) license, which permits others to distribute, remix, adapt and build upon this work, for commercial use, provided the original work is properly cited.

© 2025 by Journal of Biomedical Research.

https://doi.org/10.7555/JBR.39.20250074

Supplementary Table 2 **Primer sequences for RT-qPCR**

Genes	Forward primers (5′–3′)	Reverse primers (3′–5′)
Acta2 (Mus)	CCCAGACATCAGGGAGTAATGG	TCTATCGGATACTTCAGCGTCA
Col1a1 (Mus)	CTGGCGGTTCAGGTCCAAT	TTCCAGGCAATCCACGAGC
Gapdh (Mus)	AAGAAGGTGGTGAAGCAGG	GAAGGTGGAAGAGTGGGAGT
COL1A1 (Homo)	AGTGGTTTG GATGGTGCCAA	GCACCATCATTTCCACGAGC
ACTA2 (Homo)	CTATGAGGGCTATGCCTTGCC	GCTCAGCAGTAGTAACGAAGGA
GAPDH (Homo)	CCTTCCGTGTCCCCACT	GCCTGCTTCACCACCTTC

Supplementary Table 3 **Functional enrichment of KEGG (*P*-value < 0.05)**

Terms	Mapping	*P*-value
ECM-receptor interaction	18	0.00
RNA polymerase	5	0.00
Protein digestion and absorption	13	0.00
p53 signaling pathway	9	0.00
AGE-RAGE signaling pathway in diabetic complications	11	0.00
Amoebiasis	10	0.00
Small cell lung cancer	9	0.00
Focal adhesion	18	0.01
Human papillomavirus infection	29	0.00
Nucleotide excision repair	5	0.02
TGF-beta signaling pathway	8	0.01
Hippo signaling pathway	11	0.00
Proteoglycans in cancer	14	0.00
Relaxin signaling pathway	8	0.02
Breast cancer	9	0.02
PI3K-Akt signaling pathway	21	0.00
MicroRNAs in cancer	9	0.03
Pathways in cancer	22	0.02

Supplementary Table 4 **Swiss website target prediction top 30 results**

Target	Common name	Uniprot ID	ChEMBL ID	Target class
Renin	REN	P00797	CHEMBL286	Protease
PI3-kinase p110-gamma subunit	PIK3CG	P48736	CHEMBL3267	Enzyme
PI3-kinase p110-alpha subunit	PIK3CA	P42336	CHEMBL4005	Enzyme
Serine/threonine-protein kinase mTOR	MTOR	P42345	CHEMBL2842	Kinase
Tyrosine-protein kinase SYK	SYK	P43405	CHEMBL2599	Kinase
Acetylcholinesterase	ACHE	P22303	CHEMBL220	Hydrolase
Serine/threonine-protein kinase PIM1	PIM1	P11309	CHEMBL2147	Kinase
Histamine H3 receptor	HRH3	Q9Y5N1	CHEMBL264	Family A G protein-coupled receptor
Serine/threonine-protein kinase PIM2	PIM2	Q9P1W9	CHEMBL4523	Kinase
Serine/threonine-protein kinase PIM3	PIM3	Q86V86	CHEMBL5407	Kinase
C-C chemokine receptor type 1	CCR1	P32246	CHEMBL2413	Family A G protein-coupled receptor
Brain adenylate cyclase 1	ADCY1	Q08828	CHEMBL2899	Enzyme
Tyrosine-protein kinase receptor FLT3	FLT3	P36888	CHEMBL1974	Kinase
Tyrosine-protein kinase JAK3	JAK3	P52333	CHEMBL2148	Kinase
Tyrosine-protein kinase JAK1	JAK1	P23458	CHEMBL2835	Kinase
Tyrosine-protein kinase JAK2	JAK2	O60674	CHEMBL2971	Kinase
Tyrosine-protein kinase TYK2	TYK2	P29597	CHEMBL3553	Kinase
Macrophage colony stimulating factor receptor	CSF1R	P07333	CHEMBL1844	Kinase
Cyclin-dependent kinase 2	CDK2	P24941	CHEMBL301	Kinase
Cyclin-dependent kinase 9	CDK9	P50750	CHEMBL3116	Kinase
Epidermal growth factor receptor erbB1	EGFR	P00533	CHEMBL203	Kinase
Serine/threonine-protein kinase AKT2	AKT2	P31751	CHEMBL2431	Kinase
Multidrug resistance-associated protein 1	ABCC1	P33527	CHEMBL3004	Primary active transporter
Serine/threonine-protein kinase AKT	AKT3	Q9Y243	CHEMBL4816	Kinase
Mitogen-activated protein kinase kinase kinase 14	MAP3K14	Q99558	CHEMBL5888	Kinase
Inhibitor of apoptosis protein 3	XIAP	P98170	CHEMBL4198	Other cytosolic protein
Baculoviral IAP repeat-containing protein 2	BIRC2	Q13490	CHEMBL5462	Enzyme
Thrombin and coagulation factor X	F10	P00742	CHEMBL244	Protease
Beta-secretase 1	BACE1	P56817	CHEMBL4822	Protease
Bradykinin B1 receptor	BDKRB1	P46663	CHEMBL4308	Family A G protein-coupled receptor

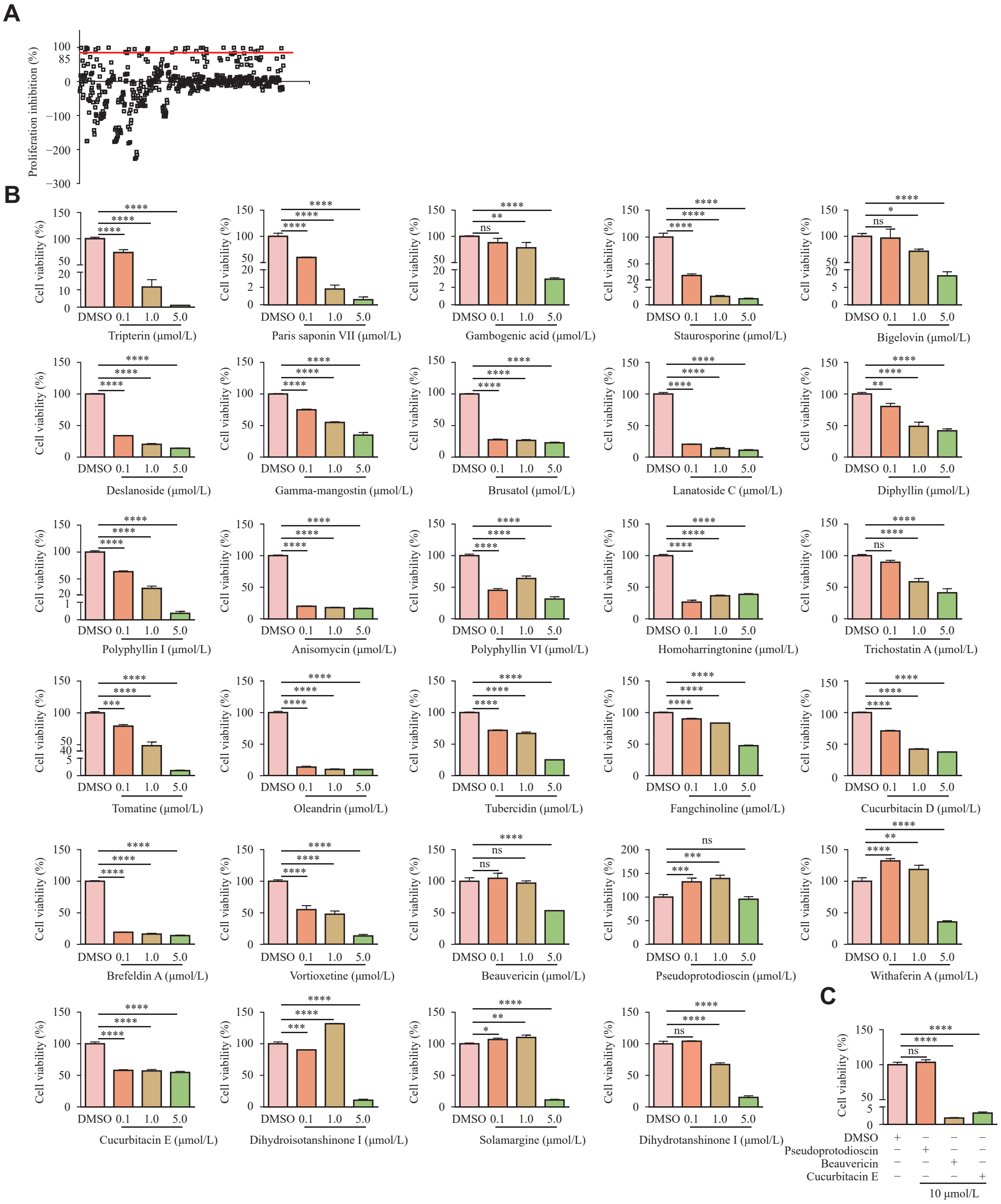

Supplementary Fig. 1 **Initial screening of compounds from natural compound library and determination of optimal concentration.** A: CCK-8 detected the inhibition rate of natural compounds on the viability of MRC-5 cells. The red line indicated that the inhibition rate was 85%, and there were 29 compounds with an inhibition rate > 85%. B: The effects of different concentrations (0.1 μmol/L, 1 μmol/L, 5 μmol/L) of natural compounds on the viability of MRC-8 cells were detected by CCK-8. Ns, no significant difference between the DMSO group. C: Effect of 10 μmol/L natural compound on the activity of MRC-5 cells. Statistical analyses were performed by two-way ANOVA with Bonferroni's test. $^{*}P < 0.05$, $^{**}P < 0.01$, $^{***}P < 0.001$ and $^{****}P < 0.000\ 1$ *vs.* the DMSO group. Abbreviations: DMSO, dimethyl sulfoxide; not significant.

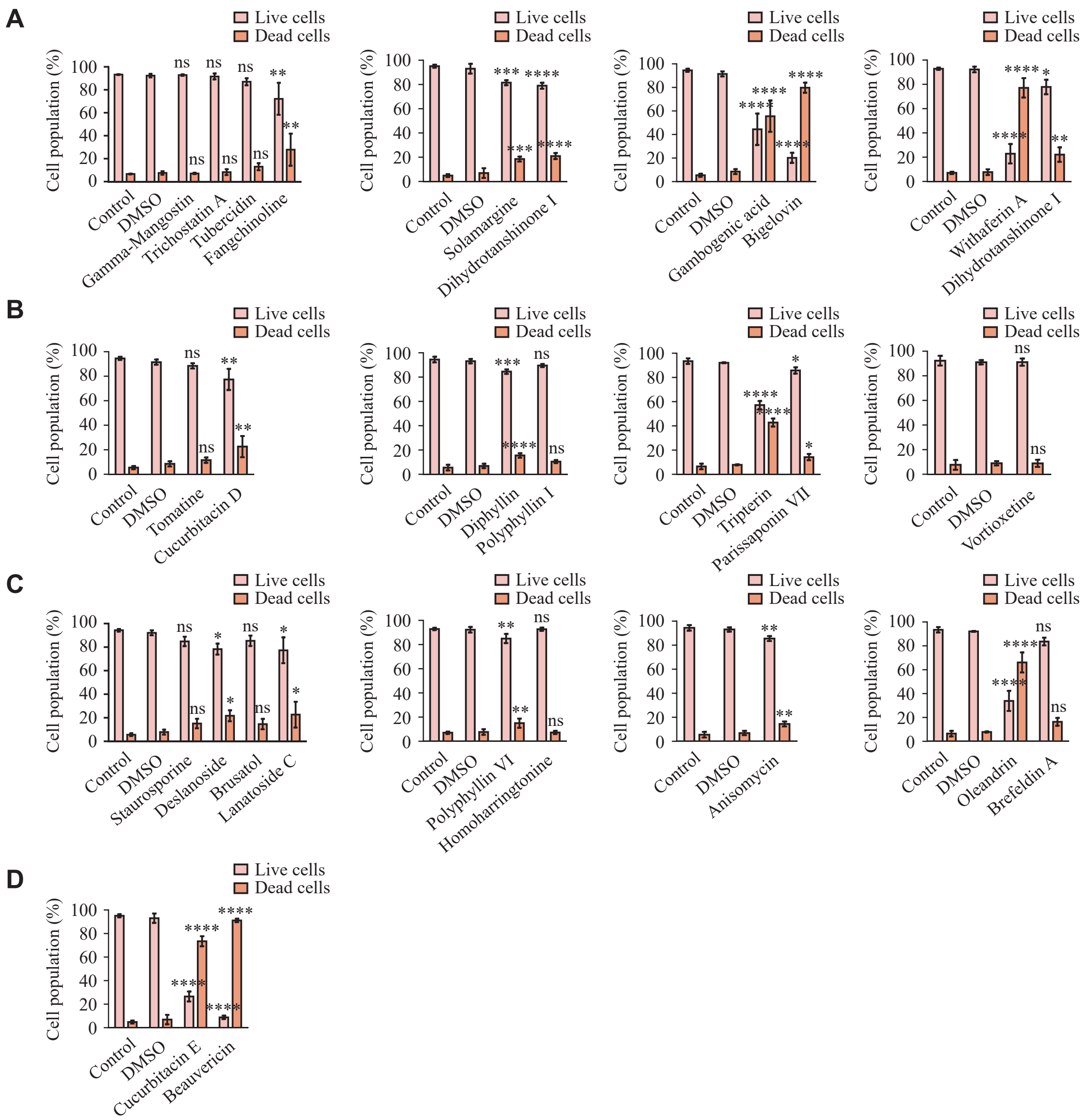

Supplementary Fig. 2 **Screen out natural compounds with cytotoxicity by trypan blue staining.** The effect of trypan blue staining on the cytotoxicity of MRC-5 at the optimum concentration (A, 5 μmol/L; B, 1 μmol/L; C, 0.1 μmol/L; D, 10 μmol/L) of natural compounds for 24 h. Statistical analyses were performed by two-way ANOVA with Bonferroni's post hoc test. $^{*}P<0.05$, $^{**}P<0.01$, $^{***}P<0.001$, and $^{****}P<0.000\,1$ *vs.* the DMSO group. Abbreviations: DMSO, dimethyl sulfoxide; ns, not significant.

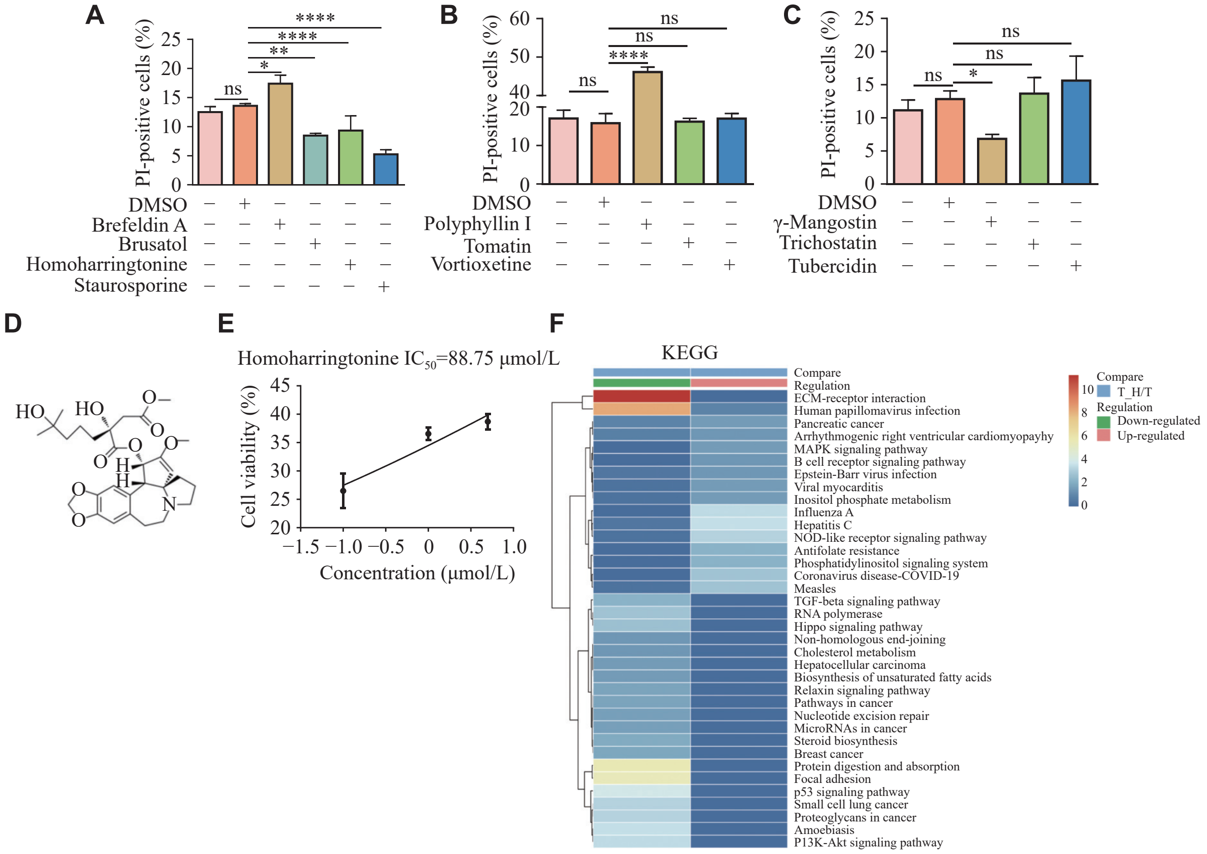

Supplementary Fig. 3 **The final candidate compound was determined as homoharringtonine by propidium iodide staining.** Flow cytometry was used to determine the proportion of PI-positive cells in MRC-5 cells treated with optimal concentrations (A, 0.1 μmol/L, B, 1 μmol/L, C, 5 μmol/L) of natural compounds for 24 h. D: Chemical structure of HHT. E: The IC_{50} value of HHT at 48 h in MRC-5 cells. F: KEGG pathway enrichment analysis of differentially expressed proteins between the TGF-β1 (left) and the TGF-β1 + HHT (right) groups. Statistical analyses were performed by two-way ANOVA with Bonferroni's post hoc test. $^{*}P < 0.05$, $^{**}P < 0.01$, and $^{****}P < 0.000\ 1$ *vs.* the DMSO group. Abbreviations: DMSO, dimethyl sulfoxide; HHT, homoharringtonine; ns, not significant.

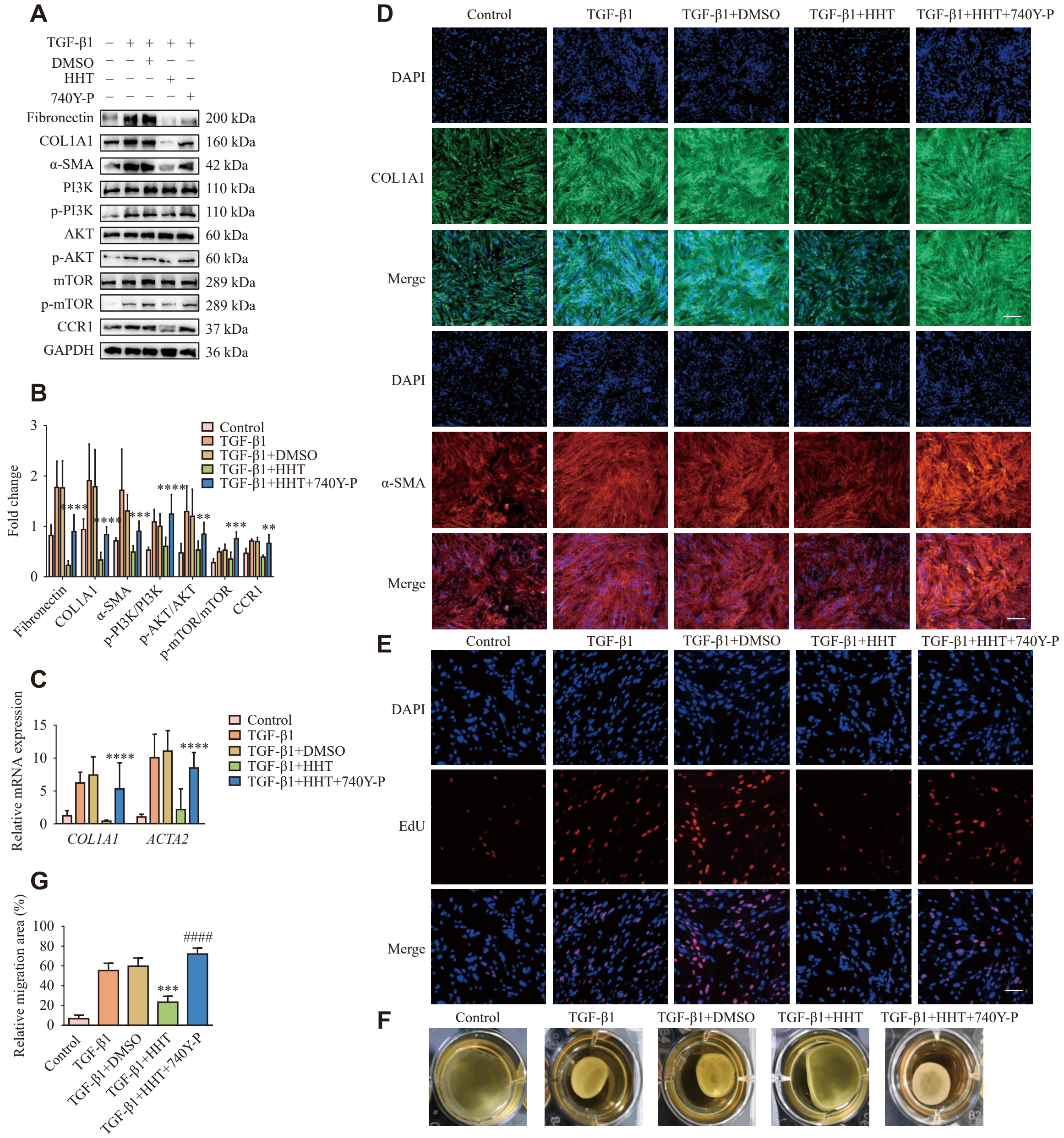

Supplementary Fig. 4 **740Y-P reversed the inhibition of homoharringtonine (HHT) on the activation and proliferation of fibroblasts through PI3K/AKT/mTOR signaling pathway.** MRC-5 cells were treated with 740Y-P, TGF-β1, and HHT simultaneously for 24 h. A: The protein levels of the PI3K/AKT/mTOR signaling pathway were compared among MRC-5 cell groups. B: Quantification of (A) (n = 3). C: Quantitative reverse transcription-PCR was used to detect the difference in mRNA levels of fibrosis marker molecules between MRC-5 cell groups (n = 4). D: The difference of fibrosis marker molecules between MRC-5 cell groups was detected by immunofluorescence staining (Scale bar, 50 μm). E: The proliferation of MRC-5 cells was detected by the EdU fluorescence staining (Scale bar, 50 μm). F: The changes in collagen contraction among 3T3 cell groups were detected by the collagen gel contraction experiment. G: Quantification of the panel F (n = 3). Statistical analyses were performed by two-way ANOVA with Bonferroni's post hoc test. $^{*}P < 0.05$, $^{**}P < 0.01$, $^{***}P < 0.001$, and $^{****}P < 0.000\,1$ *vs.* the TGF-β1+DMSO group. $^{####}P < 0.000\,1$ *vs.* the TGF-β1+HHT group. Abbreviation: DMSO, dimethyl sulfoxide.
